# Body Image Disturbances Have Impact on the Sexual Problems in Chinese Systemic Lupus Erythematosus Patients

**DOI:** 10.1155/2015/204513

**Published:** 2015-05-18

**Authors:** Biyu Shen, Yan He, Haoyang Chen, Chunmei Zhao, Li Zhu, Yingying Gao, Yunli Ren, Xueqing Wang, Jingwei Liu

**Affiliations:** ^1^Department of Nursing, The Second Affiliated Hospital of Nantong University, Nantong, Jiangsu 226001, China; ^2^Department of Rheumatology, The Second Affiliated Hospital of Nantong University, Nantong, Jiangsu 226001, China

## Abstract

SLE might affect all aspects of life including sexual functioning; previous study found that body image disturbance (BID) was the most powerful predictors of impaired partner relationships and sexual function. The current study investigated the relationship among disease parameters, quality of life, the psychological status, BID, and sexual problems in Chinese patients with SLE. A self-report survey design was administered to 168 SLE patients and 210 healthy individuals. Our results showed that 86 (55.1%) SLE patients reported impaired relationships with a sexual partner or partners, and 100 (64.1%) patients reported impaired sexual function which were significantly higher than the control group (31.6%, 35.7%, rep.). Age, marital status, depression, and BIDQ were the most powerful predictors of impaired partner relationships, while BIDQ3 and education, disease activity, and depression were the most significant causes of impaired sexual function. The study for first time reported Chinese SLE patients had sexual problems and BID was associated with sexual problems. So, early detection and interventions might not only rehabilitate the patients and their loved ones, but also improve overall health outcomes and reduce the direct and indirect costs of their medical care.

## 1. Introduction

Systemic lupus erythematosus (SLE) is a systemic autoimmune disease that disproportionately affects young women; women are affected nine times more frequently than men. About two-thirds of patients develop cutaneous manifestations that may be visible, transient, or permanent rashes, scars, depigmentation, skin dimpling, photosensitivity, and hair loss. Arthritis, serositis, nephritis, and hematological and neuropsychiatric problems are common [1]. SLE can affect different aspects of patients' life, leading to an impairment of health-related quality of life (QoL). We have reported that Chinese SLE patients have impaired QoL and psychological health problems [2]. The disease causes characteristic physical problems (i.e., chronic pain and fatigue) and emotional problems (i.e., low self-esteem and depression), and these may decrease sexual interest and reduce intercourse frequency. Partnership difficulties arising from disease-related stress can also contribute to a less active sexual life [3]. For these reasons, SLE may affect all aspects of life including sexual functioning. However, the impact of SLE on sexual problems has been less studied. Tseng et al. have reported that 52.5% have impaired sexual function in Taiwan SLE patients [4]. However, to our knowledge, few studies have focused on the sexual problems in SLE patients, especially in Chinese mainland.

Body image disturbance (BID) is commonly defined as the distortion of perceptions or cognitions related to the weight or shape of the body [5]. BID plays an important role in anxiety/depression and reduces the quality of life (QoL) [6]. It has been reported that lymphedema patients with pain have higher levels of BID and decreased sexual drive [7]. Severe BID is well documented in chronic diseases, particularly those accompanied by deformities or disabilities, such as ankylosing spondylitis (AS). Jolly et al. have reported that BID is correlated with health-related quality of life in SLE patients [8].

Recently studies have reported that vaginal pain symptoms, poorer body image, and fatigue are independent predictors of sexual dysfunction in young breast cancer survivors [9]. And sexual interest is associated with vaginal pain symptoms, body image, and weight problems. We have reported that Chinese AS patients exhibit BID, and significant relationships are found among BID, disease and psychological variables, and QoL [10]. Interestingly, we have found that disease activity and BID are the most powerful predictors of impaired partner relationships, while disease severity, BID, and physical function are the most important causes of impaired sexual function in Chinese AS patients [11]. The psychological problems, such as anxiety and depression, as well as BID occurrence, may impair sexual function and relationships. But no papers about BID and sexual problems in SLE patients have been published.

The current study examines the independent association of BID occurrence with sexual function in a Chinese population in order to provide a preliminary analysis of the clinical parameters, disease activity, physical functions, and psychological parameters associated with sexual problems in SLE patients. As a result, the factors most closely linked to a patient's sexual status can be documented and considered in the design of appropriate clinical treatment strategies.

## 2. Methods

### 2.1. Participants

SLE patients were recruited from The Second Affiliated Hospital of Nantong University from January 2010 to July 2011. A total of 170 SLE patients and 210 healthy individuals were consecutively invited to participate in a single-center cross-sectional study. Healthy individuals were used as the control group. All patients fulfilled the 1997 American College of Rheumatology (ACR) revised criteria for the classification of SLE. Patients were excluded based on the following conditions. (1) They did not complete the questionnaire; (2) they had comorbidities (e.g., serious infections or cardiac, respiratory, gastrointestinal, neurological, or endocrine diseases) that could influence SLE activity. This study was approved by the Ethics Committee of The Second Affiliated Hospital of Nantong University, and written informed consent was obtained from all participants.

### 2.2. Measures of Clinical Variables

#### 2.2.1. The Revised Self-Rating Anxiety Scale (SAS) [12]

SAS was used to evaluate the level of anxiety-related symptoms during the week prior to the survey. This self-administered test had 20 questions, with 15 items reflecting increasing anxiety levels and 5 questions reflecting decreasing anxiety levels. Each question was scored on a scale of 1 to 4 (rarely, sometimes, frequently, and always, resp.). The scores ranged from 20 to 80. Scores greater than 70 suggested severe anxious symptoms, scores between 60 and 69 indicated moderate to marked anxiety, scores between 50 and 59 suggested minimal to mild anxiety, and scores less than 50 indicated no anxious symptoms.

#### 2.2.2. The Revised Self-Rating Depression Scale (SDS) [13]

SDS was a 20-item questionnaire designed to assess mood symptoms over the past week (e.g., “I feel downhearted, blue and sad”). Each item was scored on a Likert scale ranging from 1 to 4; scores greater than 70 suggested severe depressive symptoms, scores between 60 and 69 indicated moderate to marked depression, scores between 53 and 59 suggested minimal to mild depression, and scores less than 53 indicated no depressive symptoms.

#### 2.2.3. Measure of the Quality of Life [14]

The patient's general health status was measured using the Short Form- (SF-) 36 questionnaires, which measured eight multi-item dimensions: physical functioning (PF, 10 items); role limitations due to physical problems (RP, 4 items); role limitations due to emotional problems (RE, 3 items); social functioning (SF, 2 items); mental health (MH, 5 items); energy/vitality (VT, 4 items); body pain (BP, 2 items); and general health perception (GH, 5 items). For each dimension, item scores were coded, summed, and transformed on a scale from 0 (worst possible health state measured by the questionnaire) to 100 (best possible health state).

### 2.3. Clinical Measurement of Disease Activity

The Systemic Lupus Erythematosus Disease Activity Index (SLEDAI) was used to measure disease activity [15].

### 2.4. Questionnaire Design

#### 2.4.1. Body Image Assessment

Body image was assessed using the Body Image Disturbance Questionnaire (BIDQ) containing seven scaled items scored from 0 (not affected) to 8 (extremely affected) pertaining to appearance-related concerns (BIDQ1); mental preoccupation (BIDQ2); emotional distress (BIDQ3); social, occupational, or functional impairment (BIDQ4); social life interference (BIDQ5) and educational, occupational, or other functional interferences (BIDQ6); and behavioural avoidance (BIDQ7), as previously described. Average Cronbach's alpha value is 0.82 [16, 17].

The questionnaire design was based on modified questions of numbers 13 and 14 of the Body Image Questionnaire provided in the digital form by the King's College of London, UK (http://psychology.iop.kcl.ac.uk/cadat/questionnaires/BIQ.pdf). Briefly, the questionnaire first required participants to report the magnitude of the effect of their current emotional status on sexual relationship(s) with current partners (Cronbach's *α* = 0.73). Secondly, participants were required to indicate the impact of their current status on sexual function, including sexual enjoyment and frequency (Cronbach's *α* = 0.84). For each question, participants provided ratings based on a 9-point scale ranging from 0 (not at all) to 8 (extremely).

### 2.5. Questionnaire and Measurement Administration

Questionnaires and other assessments were administered to participants from January 2010 to July 2011. Written questionnaires were provided on papers, and all participants completed the questionnaire under physician's supervision in a clinical setting. SLEDAI was evaluated by the same clinician for all patients. Nurses counted the results. The results were added to a computer database by 2 research assistants and double-checked against the original data prior to analysis.

### 2.6. Statistical Analysis

All data were expressed as means ± SD for continuous variables and as frequencies (%) for categorical variables. The statistical package was included in the STATA v.10.0 (StatCorp, USA) software for all data management and analysis. Descriptive analyses were performed to investigate participant characteristics. Student's* t*-tests were applied to assess parametric variables of independent groups, while Spearman's correlation analysis was used to assess the correlation of parametric variables. Stepwise regression analyses were conducted for sexual problems and SF-36 scores separately in order to identify significant predictors of dysmorphic concern. A *P* value less than 0.01 or less than 0.001 (*P* < 0.01 or *P* < 0.001) was considered highly statistically significant, while *P* value less than 0.05 was considered statistically significant (*P* < 0.05).

## 3. Results

### 3.1. Characteristics of SLE Patients

A total of 170 patients met the eligibility criteria. 8.236% (*n* = 14) did not complete the full questionnaire due to the lack of interest, resulting in the enrollment of 156 eligible SLE patients.  Table 1 presented the baseline participant characteristics included in our analysis. Ages ranged from 18 to 60 and the average was approximately 32.9 (SD = 10.2). The majority of our study participants were female (91.2%), married (80.76%), and low-income (64.10%) and had received less than high school education (55.12%). The SLEDAI score of participants ranged from 2 to 55 (mean = 11.8, SD = 9.5). 32 (20.51%) SLE patients were at high risk for anxiety, and 52 (33.33%) exhibited signs of depression. Comparatively, only 14 (7.1%) healthy individuals had high risk for anxiety, and 28 (14.3%) exhibited signs of depression.

### 3.2. Sexual Status in SLE Patients

There were significant differences in sexual relationship impairment as observed between SLE patients and healthy individuals. 86 (55.1%) SLE patients reported impaired relationships with a sexual partner or partners, with an overall mean score of 1.8 ± 2.0 in SLE patients on the 0–8 scale. Comparatively, 62 (31.6%) healthy individuals reported impaired relationships with a partner with an overall mean score of 1.2 ± 1.3 in SLE patients. 100 (64.10%) patients reported impaired sexual function. The frequency was significantly higher than healthy individuals (70, 35.7%) (*P* < 0.001). The overall mean scores for SLE patients regarding sexual function on the 0–8 scale were also significantly higher compared to those of healthy individuals (2.6 ± 2.7 versus 1.7 ± 2.0, resp.) (Table 2).

### 3.3. Associations between Sexual Problems and Overall Variables

The effect of SLE on sexual partner relationships showed significant correlation with scores for age (*P* = 0.01), marital status (*P* = 0.03), menstrual history (*P* = 0.04), appearance-related concerns (*P* = 0.02), distress (*P* = 0.01), impairment in social functioning (*P* = 0.003), and impairment in social life (*P* = 0.009). The effects of SLE on sexual function were significantly associated with scores of education (*P* = 0.01), appearance-related concerns (*P* = 0.03), BIDQ3 (*P* = 0.006), BIDQ4 (*P* = 0.003), BIDQ5 (*P* < 0.001), and BIDQ6 (*P* = 0.00019). These findings were detailed in Table 3.

### 3.4. Stepwise Regression Analysis for Sexual Problems

Stepwise regression analyses were used to confirm the variables most significantly correlated with psychological problems. The results showed that age, marital status, depression, and BIDQ 3,5,2,1 were the most powerful predictors of impaired partner relationships (*P* < 0.05) (Table 4). In contrast, BIDQ3 and education, disease activity, and depression were the most significant causes of impaired sexual function (*P* < 0.05) in SLE patients (Table 5).

## 4. Discussion

The associations among demographics, disease-related variables, psychological problems, BID, and sexual problem in Chinese SLE patients are examined, revealing that these SLE patients are much more likely to have impaired sexual health and partner relationships than their healthy counterparts. The current study is novel in that it assesses these parameters in a group representing the Chinese SLE population. These findings should be considered in clinical settings, where sexual health is often overlooked during the treatment of physical symptoms.

The underlying cause of disturbed sexual function in SLE patients is multifaceted, though the physical aspects are most easily assessed [18]. The physical symptoms of impaired function, pain, fatigue, and stiffness as well as psychological responses to chronic disease, such as depression and insecurity, can all contribute to sexual dysfunction [3]. Ryan et al. have reported that pain, fatigue, and stiffness may interfere with normal sexual functions [19]. SLE has been associated with substantial medical morbidity resulting in physical and occupational disability [3]. In a study by Boomsma et al., 20% of SLE patients believe that their illness would drive their family apart or worsen their relationship with their partners [20]. Partnership difficulties arising from disease-related stress can also contribute to a less active sexual life [21]. Curry et al. have found that when compared with the controls, patients with SLE have a significantly higher rate of abstention, a lower frequency of sexual activity among the sexually active, diminished vaginal lubrication, poor general sexual adjustment, and more depression [18]. Greater vaginal discomfort or pain during intercourse and difficulty in penetration due to vaginal tightness are also found. Liang et al. have reported 16% of 74 patients have sexual difficulties [22]. Stein et al. have demonstrated 10.5% of 120 patients have mild and 4% have major sexual problems [23]. Depression is a major cause of reduced quality of life in SLE patients of both genders [24]. It has been reported that depression may be the principal factor contributing to sexual dysfunction in SLE patients [25]. Questionnaires, as applied in the current study, have been shown to be the effective methods of psychological and sexual health [26]. The current study has founded that marital and education status may contribute to the sexual function of lupus. These findings are consistent with current results.

BID may adversely affect quality of life and result in psychosocial consequences, such as depression, social anxiety, impaired sexual functioning, and poor self-esteem. The effect has been previously demonstrated in body dysmorphic conditions and eating disorders, and it may also affect SLE patients with significant and chronic physical impairments [27]. Alba and Kes have reported the relation between body image and women's sexuality and the sexuality in women with eating disorders [28]. Body image is significantly altered in Tunisian nonmetastatic breast cancer patients with sexual dysfunction [29]. This study indicates that BID has impact on the sexual problems. Previous studies have found that SLE patients report poor body image [30]. Similarly, we have found that Chinese SLE patients also have poor body image which is inversely correlated with depression (data not shown). Despite these initial indications, few published studies address the correlation between BID and sexual problems in SLE patients. The current results show that, except for “consequential behavioral avoidance,” all other aspects of BID are significantly associated with impaired relationships with sexual partners. Sexual problems are shown to be common in Chinese SLE patients. The results of stepwise regression analysis in the current study demonstrates that age, marital status, and BID are most closely linked to impairments in the relationships of SLE patients and their sexual partners while BID and education are most closely linked with impaired sexual functioning in SLE patients. Interestingly, the current study reports that distress along with impairments in social functioning and social life correlates significantly with impaired sexual function. This suggests that the link between physical function and psychological factors may play a role in the sexual health and perhaps even the overall quality of life of SLE patients. Further studies will be required to assess whether psychological factors progress over time in Chinese SLE patient or if these symptoms have direct relationships with physical variables in such patients.

This study has several limitations. First, the single-center study design may mean results are not necessarily generalizable to a broader population. Second, BID and sexual problem are not separately analysed in men and women. This is a necessary next step as men and women have a dichotomy of physical build and self-perception of fitness. Third, current treatments of SLE, such as prednisone and toxic immunosuppressive drugs, may adversely affect body image. The study did not focus on the relationship between drug side effects and BID. Finally, psychological factors and sexual problem were analyzed with self-report questionnaires. Thus, further exploration of SLE patients' BID based on age, gender, and disease severity will be required to comprehensively supervise clinical prognostic guidelines, and further analyses should be conducted with assessment instruments weighted for use in SLE patients.

In summary, the study for first time has reported Chinese SLE patients have sexual problem and BID is associated with sexual problems. So, early detection and interventions may not only rehabilitate the patient and their loved ones, but also improve overall health outcomes and reduce the direct and indirect costs of their medical care. Discussions on changes in body image and the possible effects on patients with SLE need to be encouraged from both sides, by the patients and the physicians. This requires a major shift in the way we assess and provide medical care for these patients. Patients' beliefs that “doctors care for your body but do not care how you feel about your body” should challenge our approach towards the care of these patients. This requires interdisciplinary health care research, clinical collaboration, and, above all, shifting from a biomedical model to a biopsychosocial model. Increased awareness of the physical and psychological factors will aid rheumatologists and nursing specialists in initiating proper management of this subgroup of SLE patients. Sexual health may be improved by treating BID along with the physical symptoms of SLE.

## Figures and Tables

**Table 1:** Demographic and psychological and disease characteristics in SLE patients and controls.

Variables	SLE patients (*N* = 156)	Control subjects (*N* = 196)	*P*
Female gender^a^	142 (91.2)	176 (89.8)	0.75
Age, years^b^	32.9 ± 10.2	35.0 ± 11.4	0.19
SAS (≥50)^a^	32 (20.51)	14 (7.1)	<0.01
SDS (≥53)^a^	52 (33.33)	28 (14.3)	0.003
SLEDAI	11.8 ± 9.5		
Marital status^b^			
Single	30 (19.23)	56 (18.6)	0.20
Married	126 (80.76)	140 (71.4)	
Education^b^			
<9 years	86 (55.12)	76 (49.0)	0.46
≥9 years	70 (44.87)	100 (51.0)	
Work status^b^			
Working	30 (19.23)	44 (22.5)	0.58
Unemployed	126 (80.77)	152 (77.5)	
Income/person^b^			
≤2000 yuan	100 (64.10)	118 (60.2)	0.68
>2000 yuan	56 (35.90)	78 (39.8)	
Menstrual history^b^			
Normal	95 (66.90)	102 (58.0)	0.25
Abnormal	47 (33.10)	74 (42.0)	

^a^Mean ± SD; ^b^number (percentage). SLE: systemic lupus erythematosus; SAS: revised Self-Rating Anxiety Scale; SDS: revised Self-Rating Depression Scale; SLEDAI: Systemic Lupus Erythematosus Disease Activity Index.

**Table 2:** The sexual status in SLE patients in China.

SLE patients (*N* = 156)	Control subjects (*N* = 196)	
86 (55.1)	62 (31.6)	<0.001^***^
1.8 ± 2.0	1.2 ± 1.3	0.0008^***^
100 (64.1)	70 (35.7)	<0.001^***^
2.6 ± 2.7	1.7 ± 2.0	0.0004^***^

^***^ *P* < 0.001.

**Table 3:** Relationships between psychological scores, disease parameters, and sexual problems in SLE patients.

	Partner relationships		Sexual functions	
	*r*	*P*	*r*	*P*
Age	0.29	0.01^*^	0.06	0.59
Sex	−0.19	0.09	−0.03	0.80
BMI	0.04	0.70	−0.07	0.55
Marital status	−0.25	0.03^*^	0.1	0.39
Education	−0.22	0.06	−0.28	0.01^*^
Work status	−0.06	0.59	0.07	0.53
Income/person	−0.12	0.29	−0.07	0.57
Menstrual history	−0.24	0.04^*^	−0.05	0.74
SLEDAI	0.24	0.04^*^	0.29	0.02^*^
SAS	0.09	0.42	0.1	0.36
SDS	0.21	0.045^*^	0.21	0.048^*^
BIDQ1	−0.27	0.02^*^	−0.25	0.03^*^
BIDQ2	0.33	0.003^**^	0.18	0.12
BIDQ3	0.29	0.01^*^	0.3	0.006^**^
BIDQ4	0.33	0.003^**^	0.33	0.003^**^
BIDQ5	0.29	0.009^**^	0.37	<0.001^***^
BIDQ6	0.19	0.09	0.38	<0.001^***^
BIDQ7	−0.14	0.20	−0.08	0.51

^*^ *P* < 0.05;  ^**^ *P* < 0.01;  ^***^ *P* < 0.001.

SLE: systemic lupus erythematosus; BMI: Body Mass Index; SLEDAI: Systemic Lupus Erythematosus Disease Activity Index; SAS: revised Self-Rating Anxiety Scale; SDS: revised Self-Rating Depression Scale.

**Table 4:** Stepwise regression analyses of medical and psychological variables and their relationship to partner relationships in SLE patients.

Partner relationships	Coef.	SE	*t*	*P*	95% CI
Age	0.07	0.03	2.73	0.009	0.02, 0.12
Marital status	−1.44	0.63	−2.28	0.027	−2.7, 0.2
SDS	0.77	0.46	2.86	<0.001	0.45, 0.89
BIDQ3	0.22	0.09	2.33	0.024	0.03, 0.40
BIDQ5	0.27	0.12	2.30	0.027	0.03, 0.52
BIDQ2	0.28	0.11	2.55	0.014	0.06, 0.50
BIDQ1	−0.37	0.10	−3.78	<0.001	−0.57, −0.17
_cons	1.19	0.17	2.41	0.034	2.1, 2.5

**Table 5:** Stepwise regression analyses of medical and psychological variables and their relationship with sexual functions in SLE patients.

Sexual functions	Coef.	SE	*t*	*P*	95% CI
Q3	0.51	0.19	2.63	0.011	0.12, 0.90
Education	−1.40	0.69	−2.04	0.047	−2.79, −0.02
SLEDAI	0.55	0.37	2.91	0.003	0.37, 0.68
SDS	0.25	0.19	2.53	0.034	0.14, 0.51
_cons	3.43	1.21	2.83	0.007	1.00, 5.9

SLE: systemic lupus erythematosus.

SLEDAI: Systemic Lupus Erythematosus Disease Activity Index; SDS: revised Self-Rating Depression Scale.

## Abbreviations

SLE: Systemic lupus erythematosus
BID: Body image disturbance
BIDQ: Body Image Disturbance Questionnaire
SLEDAI: Systemic Lupus Erythematosus Disease Activity Index
SAS: Self-Rating Anxiety Scale
SDS: Self-Rating Depression Scale.
